# Role of shearing wave elastography in detecting early diabetic nephropathy

**DOI:** 10.1007/s11845-025-03897-5

**Published:** 2025-02-06

**Authors:** Amr M. Shaker, Shaza Y. Sleem, Mayssa I. Aly, Asmaa H. Habib, Mona G. Hassan

**Affiliations:** 1 Department of Internal Medicine and Nephrology, Kasr Al Aini Hospital, Cairo University, Al-Saray St., El-Maniel, Cairo, 11562 Egypt; 2 Department of Radiology, Kasr Al Aini Hospital, Cairo University, Cairo, Egypt

**Keywords:** Diabetic nephropathy, Kidney, Resistivity indices, Shear wave elastography, Stiffness

## Abstract

**Background:**

Diabetes mellitus is one of the systemic diseases affecting the kidneys that eventually develop end-stage kidney disease. Shear wave elastography (SWE) is a reliable and non-invasive ultrasonography test used to determine tissue elasticity. The aim of this study is to detect early diabetic nephropathy by measuring renal stiffness using shear wave elastography (SWE), renal resistivity indices, and laboratory findings in DN patients.

**Methods:**

Shear wave elastography and color duplex sonography assessments were performed in 60 diabetic nephropathy patients (divided according to eGFR into 3 stages: stage I-II-III diabetic nephropathy with equal groups of 20 patients in each stage) and 20 healthy age-matched control subjects. The SWE-derived mean value of the tissue stiffness, given in kilopascals (kPa), was correlated to patients’ clinico-laboratory data (serum creatinine and eGFR) and resistive index.

**Results:**

There is a statistically significant increase in SWE and RI in the diabetic group than control group and a statistically significant increase in SWE (mean) in CKD stage II and III when compared with CKD stage I, SWE can be used to discriminate between diabetic groups and control group at a cutoff level of > 10.5 (kPa), and also, SWE (mean) can be used to discriminate between CKD stage II and III patients at a cutoff level of > 41 (kPa), with 66.7% sensitivity and 64.9% specificity.

**Conclusion:**

Shear wave elastography is a sensitive, non-invasive, and specific diagnostic tool for the detection of diabetic nephropathy and differentiation between different stages of DN.

## Introduction

Diabetic nephropathy (DN) is one of the common serious microvascular complications of diabetes mellitus (DM). Diabetic nephropathy occurs in about 20 to 40% of all diabetics who eventually develop end-stage kidney disease [1]. So, the diagnosis of DN is of paramount importance for diabetic patients not only because of the consequences of renal progression but also because of the strong association with the risk of developing cardiovascular disease [2].

DN is characterized by albuminuria, a decrease in glomerular filtration rate (GFR), hypertension, and risk of end-stage renal diseases (ESRD) [3].

The renal resistivity indices (RI) are indices of intrarenal arteries defined as (peak systolic velocity–end-diastolic velocity)/peak systolic velocity. The normal range is 0.50–0.70. Elevated values are associated with poorer prognosis in renal disorders and renal transplant.

Resistivity indices have value in identifying diabetic patients who are developing nephropathy and can be used as an additional diagnostic tool. Also, it is well correlated with serum creatinine and albuminuria which are the biochemical parameters to diagnose DN [4].

Ultrasound shear wave elastography (SWE) is an ideal modality for assessing the alterations in various organs and diagnosing malignant tumors; thus, SWE imaging may be applied as a simple non-invasive tool for assessing the severity of chronic morphologic changes of renal parenchyma and for establishing categories of severity based on cortical stiffness measurements [5].

The aim of our study is to investigate the correlation between renal cortical stiffness (CS) measured by shear wave elastography (SWE) and clinic-laboratory data in patients diagnosed with early diabetic nephropathy stages and to compare their findings with the normal population.

## Patients and methods

The study included 20 control subjects and 60 diabetic patients, with clinico- laboratory-proven DN findings that were divided into three groups according to GFR (group I GFR > 90 ml/min/1.73*m*^*2*^, group II GFR 60–89 ml/min/1.73*m*^*2*^, group III GFR 30–59 ml/min/1.73*m*^*2*^).

The patients were referred to the ultrasound unit at Kasr Al Ainy radiology department from outpatient clinics and inpatient departments from July to December 2023. All patients were subjected to full history taking, clinical examination, laboratory investigation, and kidney assessment using color duplex sonography and SWE examination.

The study included type II diabetes mellitus patients of both sex within age 35–70 years and excluded patients more than 70 years or below 35 years, BMI > 40, GFR < 30 (calculated by CKD-EPI equation), malignancy, pregnant and lactating ladies, cardiovascular disease patients, solitary kidney, renal artery stenosis, or any renal disease other than DN. The study was approved by the ethical of Kasr Al Ainy Hospitals (MS-194–2023) and written consent was taken from all subjects.

Full history taking and examination were done and the body mass index (BMI) was assessed. Laboratory workup was done including serum creatinine, fasting blood sugar (FBS), glycated hemoglobin (HbA1C), albumin creatinine ratio in urine (ACR), and glomerular filtration rate (GFR). Abdominal ultrasound was done using an ultrasound machine (TOSHIBA Aplio 500) with (6C1) curvilinear probe for color duplex sonography to asses abdominal aorta and main renal arteries to exclude renal artery stenosis and measure peak systolic velocity (PSV), end-diastolic velocity (EDV), and resistive index (RI) in upper polar and mid and lower polar segmental renal arteries (RA) and shear wave elastography (SWE) examination to cope with the diffuse pattern of renal affection in DN interpreted in kilopascals (kPa).

### Statistical analysis

It was performed using Statistical Package for Social Sciences software (SPSS) version 23. Variables were presented as mean, standard deviation, range, numbers, and percentage. Chi-square and one-way analysis of variance (ANOVA) were used for comparison. Spearman’s correlation test was considered. *P*-value ≤ 0.05 was considered significant.

## Results

The study included 60 diabetic patients divided into 3 groups according to eGFR (stages 1, 2, and 3) including 35 males (58.3%) and 25 females (41.7%), and 20 healthy control groups included 10 males and 10 females with a mean age.

There is a statistically significant decrease in eGFR and an increase in ACR, HbA1C, and SWE in diabetic patients when compared with the control group (Table 1).

**Table 1:** Comparison between diabetic patients and control

	Diabetic (mean ± SD)	Control (mean ± SD)	*P*-value	
Age (years)	50.4 ± 11.1	45.8 ± 12	0.6	
Creatinine (mg/dl)	1.2 ± 0.57	1.0 ± 0.2	0.122	
eGFR (ml/min/1.73)	79 ± 32.3	111.5 ± 6.3	< 0.001	
ACR (mg/mg)	468.5 ± 121.5	10.2 ± 3.1	< 0.001	
HbA1C	7.5 ± 0.9	5.1 ± 0.8	< 0.001	
SWE	40.7 ± 13.2	8.2 ± 1.7		** < 0.001**

There is a statistically significant increase in ACR and serum creatinine and a statistically significant decrease in eGFR in CKD stage II and III patients when compared with CKD stage I (Table 2).

**Table 2:** Comparison of kidney function tests as regard CKD stages in diabetic group

Diabetic group		CKD stages		*P*-value
		Stage I (*N* = 20)	Stage II and III (*N* = 40)	
ACR	**Mean ± SD**	23.1 ± 3.09	468.5 ± 98.4	** < 0.001**
Creat (mg/dl)	**Mean ± SD**	0.74 ± 0.09	1.42 ± 0.57	** < 0.001**
eGFR	**Mean ± SD**	112.4 ± 9.7	55.8 ± 20.9	** < 0.001**

This table shows a statistically significant increase in SWE and RI in the diabetic group when compared with the control group. There is a statistically significant increase in SWE (mean) in CKD stage II and III cases when compared with CKD stage I cases and a statistically significant increase in SWE (mean) and RI in CKD stage III when compared with CKD stage II (Table 3).

**Table 3:** Comparison of SWE and RI in the study

	Diabetic (*N* = 60)	Control (*N* = 20)	*P*-value	Stage I CKD (*N* = 20)	Stage II and III CKD (*N* = 40	*P*-value	Stage II CKD (*N* = 20)	Stage III CKD (*N* = 20	*P*-value
SWE (mean ± SD)	40.7 ± 13.2	8.2 ± 1.7	< 0.001	38 ± 13	45.9 ± 12.2	0.027	39.6 ± 8.7	45.5 ± 13.1	< 0.001
RI (mean ± SD)	0.7 ± 0.09	0.64 ± 0.04	0.01	0.64 ± 0.05	0.68 ± 0.14	0.350	0.68 ± 0.06	0.72 ± 0.1	0.01

Using the ROC curve, it was shown that SWE can be used to discriminate between diabetic groups and control groups at a cutoff level of > 10.5 (kPa), with 100% sensitivity, 100% specificity, 100% PPV, and 100% NPV (AUC = 1.000 and *P*-value < 0.001) (Table 4)**.**

**Table 4:** Diagnostic performance of SWE in discrimination of studied groups

	Cutoff	AUC	Sensitivity	Specificity	PPV	NPV	*P*-value
SWE	> 10.5	1.000	100%	95%	100%	100%	< 0.001

*PPV* positive predictive value, *AUC* area under curve, *NPV* negative predictive value

**Fig. 1:**
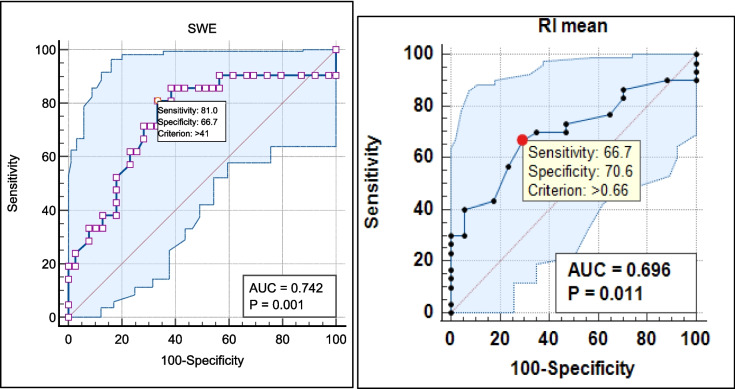
ROC curve between CKD stages as regards SWE and RI. **a** ROC curve between CKD stages as regards SWE. **b** ROC curve between CKD stages as regards RI

SWE (mean) can be used to discriminate between CKD stage II and III cases at a cutoff level of > 41 (kPa), with 66.7% sensitivity, 64.9% specificity, 64.9% PPV, and 55.2% NPV (AUC = 0.74 and *P*-value = 0.001) (Figure 1a and Table 5).

**Table 5:** Diagnostic performance of SWE and RI in discrimination of CKD stage

	Cutoff	AUC	Sensitivity	Specificity	PPV	NPV	*P*-value
SWE (mean)	** > 41**	**0.74**	**81%**	**66.7%**	**64.9%**	**55.2%**	**0.001**
RI (mean)	** > 0.66**	**0.69**	**66.7%**	**70.6%**	**80%**	**54.5%**	**0.011**

RI (mean) can be used to discriminate between CKD stage II and III cases at a cutoff level of > 0.66, with 66.7% sensitivity, 70.6% specificity, 80% PPV, and 54.5% NPV (AUC = 0.69 and *P*-value = 0.011) (Figure 1b).

**Fig. 2:**
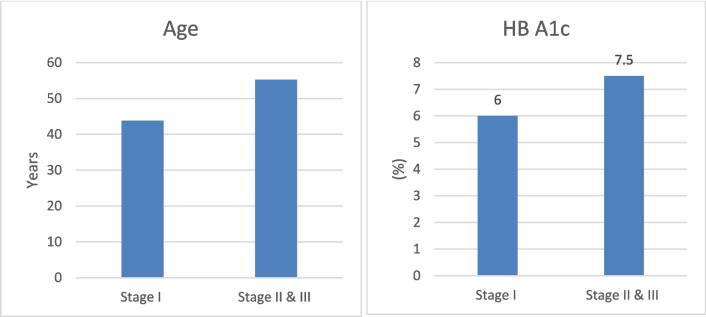
Comparison of age and HbA1C as regards CKD stages in the diabetic group

Figure 2A shows statistically significant (*P*-value = 0.001) increased age in CKD stage II and III cases when compared with CKD stage I cases. Figure 2B shows statistically significant (*P*-value = 0.002) increased HbA1C in CKD stage II and III cases when compared with CKD stage I cases.

### Multivariate regression analysis

It was done to evaluate the confusing factors that can affect our study.

The included studied factors were age, sex, A/C ratio, and HbA1C in each stage of DN (Table 6).

**Table 6:** The confusing factors that can affect our study. (a) Dependent variable: SWE. (b) Stage I, II, and III

Coefficients (a,b)							
Stage	Unstandardized Coefficients		Standardized Coefficients			95% CI for *B*	
	*B*	Std. Error	Beta	*t*	*P*-value	Lower	Upper
Stage I							
(Constant)	3.824	24.193		0.158	0.876	− 47.462	55.111
Age	− 0.186	0.421	− 0.103	− 0.443	0.664	− 1.078	0.705
Sex	8.643	6.348	0.290	1.362	0.192	− 4.814	22.100
A/C ratio	− 0.166	0.082	− 0.584	− 2.024	0.060	− 0.340	0.008
HbA1c	7.505	2.715	0.740	2.764	0.014	1.749	13.261
Stage II							
(Constant)	19.015	11.215		1.695	0.112	− 5.040	43.070
Age	0.186	0.239	0.139	0.779	0.449	− 0.326	0.698
Sex	13.374	3.965	0.683	3.373	0.005	4.869	21.879
A/C ratio	− 0.034	0.008	− 0.738	− 4.009	0.001	− 0.051	− 0.016
HbA1c	0.291	0.820	0.059	0.355	0.728	− 1.467	2.049
Stage III							
(Constant)	19.369	20.162		0.961	0.359	− 25.554	64.292
Age	0.025	0.361	0.023	0.070	0.945	− 0.778	0.829
Sex	− 3.027	3.298	− 0.196	− 0.918	0.380	− 10.377	4.322
A/C ratio	− 0.003	0.004	− 0.242	− 0.875	0.402	− 0.011	0.005
HbA1c	2.479	1.445	0.540	1.715	0.117	− 0.741	5.699

We found in stage I that only HbA1c can affect SWE measures as high levels of HbA1C parallel to increased SWE measures. In stage II, the SWE measure was inversely related to the A/C ratio; in stage III, the SWE measure was not affected by age, sex, A/C ratio, or HbA1C.

## Discussion

Diabetic nephropathy (DN) is considered a clinical and laboratory condition that is characterized by albuminuria on at least two occasions 3–6 months apart [6]. Early detection of DN can prevent the progression of the disease additionally. Optimal glycemic control can delay or prevent other complications of DM and progression to ESKD [7].

Resistivity index (RI) is the ratio of the difference between PSV and EDV flow velocities to PSV velocity as determined by spectral Doppler waveform interrogation of the intrarenal arteries. It is a simple and non-invasive approach for the evaluation of the renal functional and structural alterations in DN [8]. Shear wave elastography (SWE) is a simple and cost-effective diagnostic tool used for the assessment of tissue elasticity [9]. It measures the velocity of the transmitted shear waves through the examined tissues in meter/second (m/s), then the software processes the obtained data into a tissue stiffness parameter, and finally is interpreted in kilopascals (kPa) [10].

The study aims to evaluate the diagnostic value of shearing wave elastography in early-stage diabetic nephropathy patients and its relation to different stages of diabetic nephropathy.

We found the increase of age in diabetic patient groups positively associated with the progression of the stage of diabetic nephropathy as the age in CKD stage II and III patients is higher than CKD stage I. This was favorable with Fabre et al. [11] who said that diabetic patients with advanced age, longer duration of diabetic illness, poor glycemic control status, and co-morbid hypertension were the determinant factors of DN.

The systematic review and meta-analysis study done by Azagew et al. identifies the important risk factors for DN. The findings of this study revealed that advanced age is a risk factor for DN [12].

In our patients, high values of HbA1C levels were recorded with stage II and III compared to stage I DN. The findings also depict that glycemic control status is the determinant factor of DN. This was in agreement with Azagew et al.’s study that showed diabetic patients who have poor glycemic control status are 2.57 times more likely to develop DN compared to those diabetic patients with good glycemic control status [12]. Over time, poorly controlled diabetes can cause damage to blood vessel clusters in the kidneys that filter waste. High blood glucose affects the microvasculature of the kidneys, which results in nephron sclerosis [13].

We found that shear wave elastography is a useful method with 100% specificity and 100% sensitivity to detect diabetic group from the normal population with cutoff level 10.5 (kPa) and discriminating stage II and III at a cutoff level > 41 with 66.7% sensitivity and 64.9% specificity.

Yuksekkaya et al. proved the mean shear wave elastography values were significantly higher in the diabetic kidney disease group; they obtained statistically significantly higher shear wave elastography values in stages 2 and 3 diabetic kidney disease subjects than control subjects and in patients with stage 3 diabetic kidney disease compared to those with stage 1 diabetic kidney disease (*P* < 0.05 for all). They obtained a cutoff value of 9.23 kPa for predicting diabetic kidney disease in early stages, with a sensitivity of 67% and a specificity of 82% [14].

In a prospective study by Chen et al. (2024), 162 patients underwent renal SWE examinations and renal biopsies, and combined eGFR and SWE value could improve diagnostic performance in distinguishing between mild renal fibrosis and moderate-to-severe renal fibrosis in patients with CKD. The SWE value displayed a sensitivity of 84.1% and a specificity of 62.2% [15].

Similar to our results, Liu et al. reported increased SWE values in the early (7.93 kPa) and middle stages (16.88 kPa) of patients with DN compared to the diabetic subjects without nephropathy (5.51 kPa) [16]. Koc et al. reported increased stiffness in DM without diabetic nephropathy compared to healthy (9.86 kPa vs. 7.92 kPa) [17]. Also, Goya et al. observed increased shear wave velocity values in DN. They obtained the highest shear wave velocity values in patients with stage 2, in contrast to our result on patients with stage 3 [18].

The study shows statistically significant increased RI in the diabetic group when compared with the control group. There is a statistically significant increased RI in CKD stage III when compared with CKD stage II.

RI (mean) can be used to discriminate between CKD stage II and III cases at a cutoff level of > 0.66, with 66.7% sensitivity, 70.6% specificity, 80% PPV, and 54.5% NPV (AUC = 0.69 and *P*-value = 0.011).

Our study is consistent with the results found by Kuttancheri et al.; they showed significantly higher renal resistive index values (mean—0.72) in diabetic than in non-diabetic CKD patients (mean—0.65) (*P* = 0.001). There was a notable negative correlation between renal resistive index and eGFR, signifying that RI progressively increased from lower to higher stages of CKD [5].

A cross-sectional study by Khairallah et al. evaluated renal RI in the DN group and non-DN group. Patients with DN had significantly higher RI (0.89 ± 0.11 vs. 0.49 ± 0.18; *P* < 0.001). At cutoff point > 0.73, the resistive index had 82.5% overall accuracy with the area under the curve being 0.837 for the prediction of diabetic nephropathy [19].

Our study was in agreement with the study of Baz et al.; their results showed a significant positive correlation between the mean resistivity indices and the mean renal cortical stiffness on one hand and the patients’ clinical-laboratory data [20].

Also, Elshweehy et al. reported that the mean RI in all stages of DN group was significantly higher than the control group and found that a progressive increase in the mean RI values was significantly associated with an increase in stages of DN (*P*-value < 0.001) [21].

Maralescu et al. proved that novel elastography methods can distinguish between individuals with different stages of renal fibrosis and correlate with renal function [22].

Chen et al.’s study showed that renal elasticity was associated with a 3.5-fold increment in the risk of moderate-to-severe renal fibrosis [23].

Cao et al. did a systematic review and meta-analysis of the 454 studies which revealed that the diagnostic performance of SWE was good in detecting mild and severe renal fibrosis and fair in moderate fibrosis. They suggested large prospective international multicenter studies in different countries are needed to compare the various imaging modalities in SWE and different etiologies of CKD to further evaluate the potential of SWE. More in-depth ultrasound imaging studies in the renal field are expected [24].

Lin et al. described an increase in renal parenchymal stiffness in the late stages of DN compared to the early ones [25].

However, Cè Felisaz et al. did a meta-analysis study that proved very heterogeneous in terms of design and results. The shear wave velocity difference of − 0.82 m/s (95% CI − 1.72–0.07) between CKD patients and controls was not significant [26].

From our current study, we may suggest shear wave elastography may be used as a noninvasive, simple, and quantitative method to provide diagnostic information as a part of routine management of patients with type 2 diabetes mellitus, especially in the early stages of diabetic kidney disease.

Our study showed that elastography values and the RI values were significantly higher in the patients with type 2 DM than in the control group. Thus, it can be concluded that it is possible to determine renal fibrosis and predict the possibility of diabetic nephropathy development with the non-invasive methods of SWE, color Doppler US, and RI without the need for biopsy.

These different methods could be used as a promising tool in the early identification of DN. Multiple future studies at multiple centers with long-term duration of assessment are warranted. The most important result of our study was the SWE evaluation of patients with type 2 DM who do not have signs of diabetic nephropathy**.**

Finally, we measured ACR in three groups of diabetic nephropathy that was undetectable in the first and second stages with few numbers of cases showing microalbuminuria in the 3rd stage of DN. A/C ratio can be used as a standard reference to estimate the severity of chronic kidney disease (CKD stages) instead of eGFR in other studies of a large number of cases, which may have contributed to renal resilience in the participants.

The limitation of the current study includes a relatively small sample size, the lack of standard cutoff values for normal and abnormal renal stiffness, and the lack of standardization of SWE examination protocol. This is a single institution study, so its results can not be generalized over the whole region. Consequently, larger multicenter studies should be conducted to define an optimal cutoff point for diagnosis of preclinical, early, and late DN.

We finally concluded that shear wave elastography is a highly sensitive, specific, and accurate diagnostic tool for the assessment of early diabetic nephropathy versus the control group, as well as differentiation between the early stage and late stage of DN.

Shear wave elastography is a useful tool when combined with laboratory and Doppler findings which increase the sensitivity and specificity of diagnosing early diabetic nephropathy when compared to the diagnostic value of renal color Doppler alone.

## Data Availability

Not applicable.
